# Determinants of COVID-19 Vaccine Engagement in Algeria: A Population-Based Study With Systematic Review of Studies From Arab Countries of the MENA Region

**DOI:** 10.3389/fpubh.2022.843449

**Published:** 2022-05-30

**Authors:** Salah Eddine Oussama Kacimi, Selma Nihel Klouche-Djedid, Omar Riffi, Hadj Ahmed Belaouni, Farah Yasmin, Mohammad Yasir Essar, Fatma Asma Taouza, Yasmine Belakhdar, Saliha Chiboub Fellah, Amira Yasmine Benmelouka, Shoaib Ahmed, Mohammad Aloulou, Abdellah Bendelhoum, Hafida Merzouk, Sherief Ghozy, Jaffer Shah, Mohamed Amine Haireche

**Affiliations:** ^1^Department of Medicine, Faculty of Medicine, University of Tlemcen, Tlemcen, Algeria; ^2^Laboratoire de Biologie des Systèmes Microbiens (LBSM), Ecole Normale Supérieure de Kouba, Algiers, Algeria; ^3^Dow University of Health Sciences, Karachi, Pakistan; ^4^Kabul University of Medical Sciences, Kabul, Afghanistan; ^5^Faculty of Medicine, University of Oran, Oran, Algeria; ^6^Faculty of Medicine, University of Annaba, Annaba, Algeria; ^7^Faculty of Medicine, University of Algiers, Algiers, Algeria; ^8^Punjab Medical College, Faisalabad, Pakistan; ^9^Faculty of Medicine, University of Aleppo, Aleppo, Syria; ^10^Laboratory of Physiology, Physiopathology and Biochemistry of Nutrition, Department of Biology, Faculty of Natural and Life Sciences, Earth and Universe, University of Tlemcen, Tlemcen, Algeria; ^11^Neurovascular Research Lab, Radiology Department, Mayo Clinic, Rochester, MN, United States; ^12^Nuffield Department of Primary Care Health Science, Medical Science Division, Oxford University, Oxford, United Kingdom; ^13^Medical Research Center, Kateb University, Kabul, Afghanistan; ^14^Research Department, Scientia Vallis, Paris, France

**Keywords:** COVID-19, vaccine, Algeria, acceptance, hesitancy, Middle-East and North African (MENA), SARS-CoV-2, immunization

## Abstract

**Background:**

The Algerian COVID-19 vaccination campaign, which started at the end of January 2021, is marked by a slowly ascending curve despite the deployed resources. To tackle the issue, we assessed the levels and explored determinants of engagement toward the COVID-19 vaccine among the Algerian population.

**Methods:**

A nationwide, online-based cross-sectional study was conducted between March 27 and April 30, 2021. A two-stage stratified snowball sampling method was used to include an equivalent number of participants from the four cardinal regions of the country. A vaccine engagement scale was developed, defining vaccine engagement as a multidimensional parameter (5 items) that combined self-stated acceptance and willingness with perceived safety and efficacy of the vaccine. An Engagement score was calculated and the median was used to define engagement vs. non-engagement. Sociodemographic and clinical data, perceptions about COVID-19, and levels of adherence to preventive measures were analyzed as predictors for non-engagement.

**Results:**

We included 1,019 participants, 54% were female and 64% were aged 18–29 years. Overall, there were low rates of self-declared acceptance (26%) and willingness (21%) to take the vaccine, as well as low levels of agreement regarding vaccine safety (21%) and efficacy (30%). Thus, the vaccine engagement rate was estimated at 33.5%, and ranged between 29.6-38.5% depending on the region (*p* > 0.05). Non-engagement was independently associated with female gender (OR = 2.31, *p* < 0.001), low adherence level to preventive measures (OR = 6.93, *p* < 0.001), private-sector jobs (OR = 0.53, *p* = 0.038), perceived COVID-19 severity (OR = 0.66, *p* = 0.014), and fear from contracting the disease (OR = 0.56, *p* = 0.018). Concern about vaccine side effects (72.0%) and exigence for more efficacy and safety studies (48.3%) were the most commonly reported barrier and enabler for vaccine acceptance respectively; whereas beliefs in the conspiracy theory were reported by 23.4%.

**Conclusions:**

The very low rates of vaccine engagement among the Algerian population probably explain the slow ascension of the vaccination curve in the country. Vaccine awareness campaigns should be implemented to address the multiple misconceptions and enhance the levels of knowledge and perception both about the disease and the vaccine, by prioritizing target populations and engaging both healthcare workers and the general population.

## Graphical Abstract

Visual summary of the structure and main findings of the study. 
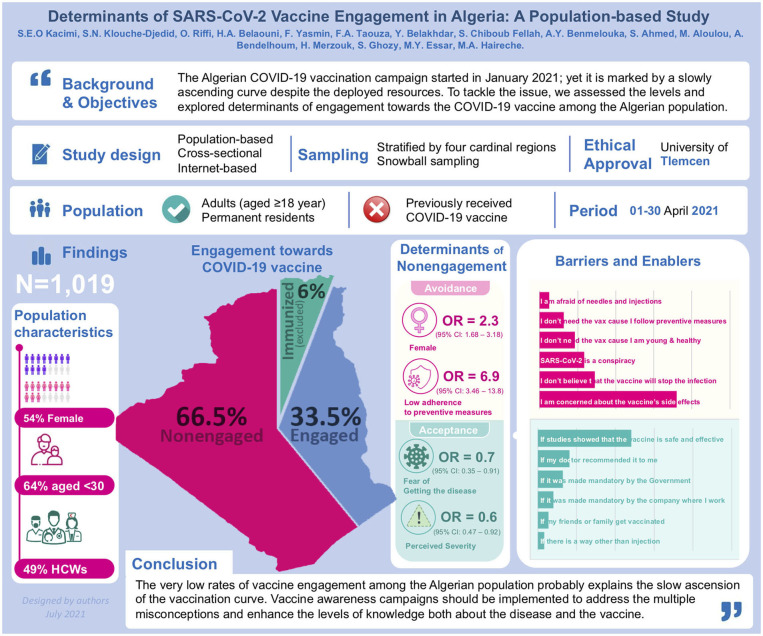

## Background

Amid the ongoing COVID-19 pandemic and the lack of effective curative treatments, mass vaccination is perceived as the only effective strategy to control the pandemic and reduce its global impact on individuals and societies. Different types of COVID-19 vaccines have been developed so far, using different techniques including mRNA, adenovirus vector, adjuvanted protein, or live-attenuated or inactivated virus vaccines. The current evidence supports the efficacy of the majority of the commercialized and recommended vaccines in eliciting robust production of neutralizing antibodies in the short- and median-term, correlating with a significant reduction in the incidence of COVID-19 infection both in the clinical trial and real life (1–4).

As of February 2022, the number of vaccine doses that have been administered globally was estimated at more than 10 billion, with nearly 60% of the world's population being fully vaccinated (5). However, there is a great discrepancy in vaccination rates between the industrialized countries such as Canada (212.6 doses per 100 population), the United Kingdom (205 doses per 100 population), and the European countries, and developing and low-income countries such as Algeria (31.1 doses per 100 population), Egypt (69.7 doses per 100 population), and Sudan (13.0 doses per 100 population) (5, 6). The COVID-19 Vaccines Global Access (COVAX) initiative's campaign efforts to finance and distribute the vaccine in poor countries are limited by multiple factors including the difficulty of providing all the needs of these countries and the limited funding sources (7). On the other hand, the recent emergence and spread of novel viral variants, notably the B.1.1.7 (Alpha), B.1.351 (Beta), P.1 (Gamma), B.1.617 (Delta), B.1.617.2 (Delta-plus), B.1.525 (Eta), B.1.429 (Epsilon), and B.1.1.529 (Omicron) variants compromised the forecasted transition, in the short run, to the pre-pandemic normal life (8–12). As a consequence, the resolution of the issue depends on a three-fold concern, including the success of the global mass immunization, the long-term efficacy of the vaccines, and the dreaded scenario of resistance of the emerging variants to the vaccine-induced immunity (13–15).

In addressing the determinants of success for this global strategy, people's engagement to local vaccination campaigns constitutes a major determinant, besides the adherence to prevention policies and recommendations. Although the modern experience with mass vaccination proved to be effective in controlling and eradicating outbreaks such as Polio, Smallpox, and other diseases (16), vaccine hesitancy has long been identified as one of the major threats facing global health (17–19). Due to several factors, the COVID-19 vaccine is subject to recurrent popular misconceptions and uncertainties, which constitutes further barriers to public adherence to the vaccination strategy (20). Such misconceptions are reported to be particularly prevalent in developing countries and conservative societies, associated with high rates of vaccine hesitancy (21). Consequently, substantial discrepancies have been observed in vaccine acceptance rates across the different regions and cultures (22), with remarkably higher vaccine hesitancy in Eastern Europe, North Africa, the Middle-East, and Central Asia (23).

In Algeria, the largest African country and the 9th country in Africa in terms of population size, the fight against the virus has gone through successive phases since the first confirmed case was declared on February 25, 2020. Since the early phase of the pandemic, the Algerian government opted for broad travel cancellations combined with the intermittent implementation of restrictive and semi-restrictive measures locally, in addition to the deployment of tremendous healthcare resources to treat the infected population (24–26). As of 21 May 2021, date of start of the current study, the country has recorded 126,434 confirmed cases and 3,405 deaths (27). In March 2022, date of last revision of the paper, these figures have doubled with 265,346 confirmed cases and 6,860 deaths (28). The national vaccination campaign started by the end of January 2021 and the current local policy targets all vulnerable groups. However, the vaccination rate remains remarkably low, reaching only 2.5 million doses by 14 July 2021, which represented a coverage rate estimated at 5.8% of the population (6, 29). To date, i.e., 10 March 2022, the coverage rate remains low with only 15% of the population being fully vaccinated (28). This represents a concern, contrasting with the country's efforts to promote the vaccination.

In an attempt to explain this low vaccination rate, the present study was designed to evaluate the levels of engagement among Algerians toward the COVID-19 vaccine and to analyze the associated sociodemographic factors. Additionally, it explored the associated misconceptions and eventual barriers and enablers of vaccine acceptance. Such data would assist the decision-makers in implementing strategic amendments on the vaccination policy and the related communication approaches. We further conducted a systematic review on vaccine acceptance in the Arab countries of the Middle-East and North African (MENA) region.

## Methods

### Cross-Sectional Study

#### Design & Population

A nationwide online-based cross-sectional study was conducted among the general population of Algeria, between March 27 and April 30, 2021. It involved adult (aged 18 years and older) males and females of all regions, who were permanently residing inside the country during the study period. Since the study aimed to understand the contribution of non-engagement to vaccine in explaining the low vaccination rates, individuals who had previously received the COVID-19 vaccine were excluded. The study was approved by the institutional review board of the University of Tlemcen [14/2021 EDCTU]. All participants provided informed consent prior to their participation.

Algeria is a North African republic, on the Mediterranean Sea, whose capital is Algiers. It has a population estimated at 45.2 million, 73% of them living in urban areas, mainly in the north of the country. Algerian population is considered young with a median age of 28.5 years and a total fertility rate is estimated at 3.1 live births per women (30).

#### Sample Size and Sampling Technique

The sample size (*N* = 385) was calculated using the single proportion sample size calculation formula, to detect an unknown vaccine acceptance rate (*P* = 50%) with 95% confidence interval (95%CI), 80% statistical power, and 5% margin error, among the total Algerian population. According to the WorldOMeter estimates, based on the United Nations data, the Algerian population was 44,594,368 as of May 30, 2021 (31).

A two-stage stratified, non-probability snowball sampling method was used in this study. In Stage 1, Algeria was divided into four cardinal regions (strata) including North/Center, East, West, and South. In stage 2, participants who were directly reached by the investigator were solicited to disseminate the questionnaire among their acquaintances until reaching a comparable number (~N/4) of participants in each region (stratum).

#### Instrument Development and Validation

The questionnaire used in the present study was designed based on previously published papers related to vaccine acceptance (32–36). It was developed in English and translated into the Arabic language by a native speaker, considering the vocabulary specificities of the Algerian population (Supplementary Material). The final questionnaire was administered in Arabic and comprised the following 5 mandatory sections:

1. Sociodemographic data: including participant's age, gender, marital status, residency region, monthly income in Algerian Dinars (AD), educational level, occupation, living mode (alone or with family), children (yes or no), and living area (rural or urban); and whether the participant has a chronic disease or lives with someone with a chronic disease.
2. Health perception: including perceived health status (1 item) and perception about COVID-19 as an illness (3 items) including the perceived probability of contracting COVID-19 infection, level of fear of being infected, and perceived severity of COVID-19.
3. Levels of adherence to government recommendations and preventive measures against COVID-19: including 7 items, such as social distancing, hand cleaning, care-seeking behavior in case of suggestive symptoms, etc. Each of the 7 items was formulated as a Likert-type agreement scale with 5 levels, including “Strongly Disagree (score = 1),” “Disagree (2),” “Neutral (3),” “Agree (4),” and “Strongly agree (5)”.
4. Attitudes and beliefs toward COVID-19 vaccination: including the 5 following items: “I think that COVID-19 vaccination is effective”; “In principle, I accept to get the COVID-19 vaccination”; I will receive the COVID-19 vaccination as soon as possible whenever it is available”; “I think that the best way to avoid the complications of COVID-19 is by being vaccinated”; “I think that COVID-19 vaccination is safe”. A 5-score Likert-type agreement scale was used to encode the answers from “Strongly disagree (score = 1)” to “Strongly agree (score = 5).”
5. Barriers and enablers of COVID-19 vaccine acceptance: including a predefined list of potential factors that may negatively (barriers) or positively (enablers) impact the participant's decision to receiving the COVID-19 vaccine. The list comprised 6 barriers such as concerns regarding vaccine's side effects, conspiracy theory beliefs, etc., and 6 enablers such as vaccination enforcement policy, recommendation by a physician, etc.

The questionnaire sections and items underwent face and content validity by the research team members, with the help of two public health and epidemiology experts. Further, the questionnaire was administered in a pilot sample (*n* = 31) to assess the clarity and full understanding of questions and items. Data collected from the pilot sample was not used in the final analysis. A copy of the Arabic or English questionnaire is available upon request from the first or corresponding author.

#### Data Collection Procedure

The final, validated version of the questionnaire was edited as an online survey in Google Forms, where all items were set to “mandatory” mode. An introduction was embedded in the first page of the survey consisting of the study description, an informed consent agreement, and one question related to previous COVID-19 vaccination history (eligibility criterion). The online survey link was disseminated through social media platforms including Facebook, WhatsApp, and Messenger. Additionally, we distributed the survey link through specific Facebook groups targeting healthcare workers and medical students, both regarding their enrollment and to enhance the snowball sampling. No incentive was offered for participation or data collection. Data collection was anonymous and identity collecting options of Google Forms were deactivated. We followed the Strengthening the Reporting of Observational Studies in Epidemiology (STROBE) statement for reporting this study (37).

#### Statistical Methods

##### Score Calculation and Outcome Definition

Engagement score, the primary outcome, was calculated by summing the scores of the 5 items (Supplementary Table 1) from efficacy, prevention of complications, safety, acceptance, and willingness subscales; high scores indicated higher levels of engagement to the vaccination. The use of an engagement score was based on the assumption that actual engagement to the vaccine is a multidimensional concept depending on the participant's perceptions and attitudes toward the vaccine safety, efficacy, prevention from complications (items 1, 4, and 5), and declared acceptance and willingness to receive it (items 2 and 3).

Adherence score (range 7−35) was calculated by summing the scores of the 7 items (Supplementary Table 2) from the Adherence Level subscale; higher scores indicated higher adherence levels to recommendations and preventive measures. The variable related to adherence level was categorized into three subcategories (Low level, medium level, and high adherence level).

##### Statistical Analysis

Categorical variables were presented as frequency and percentage, while continuous variables were presented as mean and standard deviation (SD) in the descriptive statistical analyses. The Chi-square test was used to analyze the association between categorical variables. Bivariate correlations between numerical variables were tested using Pearson's correlation. Moreover, a multivariate logistic regression was used to analyze the determinants of COVID-19 vaccine's engagement. A *p* <0.05 was indicative of statistical significance. Statistical analysis was performed by means of IBM's SPSS for Windows, Version 25.0 (SPSS Inc., Chicago, IL, USA).

### Systematic Review

#### Database Search and Eligibility Criteria

**Figure 1:**
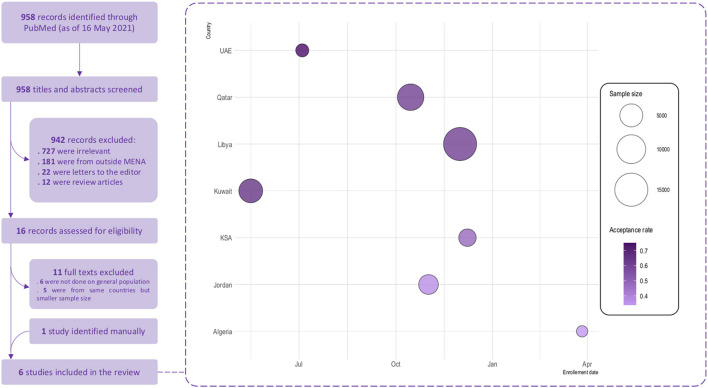
COVID-19 vaccine acceptance in the Arab countries from the MENA region–Systematic review flowchart and findings. Size of the bubbles represents the sample size used and the color gradient represents the acceptance rate of vaccination reported in each study; light colors represents lower acceptance rates and dark colors represents higher acceptance rates. Enrollement date is the starting date of data collection.

We conducted a systematic review in compliance with the Preferred Reporting Items for Systematic Reviews and Meta-Analyses (PRISMA) reporting guidelines (38). Medline was searched through the PubMed database using the following search strategy: (COVID-19 OR SARS-CoV-2) AND (vaccine OR vaccination) AND (hesitancy OR acceptance) to retrieve related studies published from the database inception to May 16th, 2021. Only studies targeting the general population and reporting COVID-19 vaccination acceptance rate and studies conducted in Arab countries of the MENA region were included. Review articles, editorials, case reports, and case series were excluded. Additionally, the reference list of included articles was scrutinized to identify extra articles (Figure 1).

#### Study Selection

Two authors independently screened titles and abstracts of retrieved articles against the inclusion/exclusion criteria. Full-texts of potentially eligible articles were further assessed by two authors for final decision. Discrepancies were resolved via discussion. In the case of multiple reports from the same country, the one containing the greatest amount of information (for example, largest sample size) was included in the review.

#### Data Extraction

Three investigators extracted data from relevant articles using a data extraction form. The collected data included the author's name, study country, study period, sampling method, sample size, percentages of males and older age, acceptance rate, the predictors for COVID-19 vaccine acceptance and avoidance. A fourth experienced investigator double-checked all collected evidence for accuracy.

#### Quality Assessment

The quality assessment of the included studies was performed using to the National Institute of Health study quality assessment tool (35).

## Results

### Sociodemographic Characteristics

A total of 1,019 respondents were included, with equal distribution across the four cardinal regions in Algeria. Of these, 545 (54%) were female, 650 (64%) were aged 18–29 years, and 500 (49%) were in the healthcare sector including medical students (36%) or healthcare professionals (13%). The majority were single (70%) and had a high educational level (84%). Regarding comorbidities, 136 (13.3%) had a chronic disease and 531 (52.1%) were living with at least one family member having a chronic disease. Otherwise, 87.0% of the participants rated their health status to be good or excellent (Table 1).

**Table 1:** Sociodemographic characteristics and answering patterns to different questionnaire scales in total population and by comparison between healthcare workers vs. medical students vs. the general population.

**Characteristics**	**Total**,	**General population**,	**Healthcare workers**,	**Medical students**,	**P-value**
	***n* (%)**	***n* (%)**	***n* (%)**	***n* (%)**	
Total	1019	519	136	364	
Age					<0.001
More than 60	54 (05%)	52 (10%)	2 (01%)	0 (0%)	
40–59	107 (11%)	99 (19%)	7 (05%)	1 (0.2%)	
30–39	208 (20%)	174 (34%)	32 (24%)	2 (1%)	
18–29	650 (64%)	194 (37%)	95 (70%)	361 99%)	
Gender					<0.001
Males	474 (47%)	306 (59%)	42 (31%)	126 (35%)	
Female	545 (54%)	213 (41%)	94 (69%)	238 (65%)	
Region					<0.001
Center	250 (25%)	146 (28%)	28 (21%)	76 (21%)	
East	257 (25%)	107 (21%)	32 (24%)	118 (32%)	
West	252 (25%)	112 (22%)	42 (31%)	98 (27%)	
South	260 (26%)	154 (30%)	34 (25%)	72 (20%)	
Area					0.651
Urban	825 (81%)	417 (80%)	114 (84%)	294 (81%)	
Rural	194 (19%)	102 (20%)	22 (16%)	70 (19%)	
Marital status					<0.001
Ever married	307 (30%)	262 (50%)	38 (28%)	7 (02%)	
Never married	712 (70%)	257 (50%)	98 (72%)	357 (98%)	
House setting					0.001
With family	962 (94%)	477 (92%)	129 (95%)	356 (98%)	
Alone	57 (6%)	42 (8%)	7 (5%)	8 (2%)	
Income					<0.001
>100K AD	199 (20%)	95 (18%)	33 (24%)	71 (20%)	
50K−100K AD	347 (34%)	157 (30%)	62 (46%)	128 (35%)	
<50K AD	473 (46%)	267 (51%)	41 (30%)	165 (45%)	
Children					<0.001
No	763 (75%)	296 (57%)	106 (78%)	361 99%)	
Yes	256 (25%)	223 (43%)	30 (22%)	3 (1%)	
Having chronic disease					<0.001
No	883 (87%)	427 (82%)	120 (88%)	336 (92%)	
Yes	136 (13%)	92 (18%)	16 (12%)	28 (08%)	
Living with someone who has a chronic disease					0.863
No	488 (48%)	246 (47%)	68 (50%)	174 (48%)	
Yes	531 (52%)	273 (53%)	68 (50%)	190 (52%)	
Perceived health status					0.023
Below average	131 (13%)	439 (85%)	126 (93%)	323 (98%)	
Good or excellent	888 (87%)	80 (15%)	10 (7%)	41 (11%)	
Fear of getting the disease					0.011
No	144 (14%)	79 (15%)	9 (07%)	56 (15%)	
Got the disease	164 (16%)	80 (15%)	33 (24%)	51 (14%)	
Yes	711 (70%)	360 (69%)	94 (69%)	257 (71%)	
Perception of COVID-19 severity					0.013
Low	439 (43%)	244 (47%)	50 (37%)	145 (40%)	
Moderate	317 (31%)	161 (31%)	50 (37%)	106 (29%)	
High	263 (26%)	114 (22%)	36 (36%)	113 (31%)	
Level of Adherence to preventive measures					0.024
Low	93 (9%)	50 (10%)	7 (05%)	36 (10%)	
Moderate	491 (48%)	245 (47%)	56 (41%)	190 (52%)	
High	435 (43%)	224 (43%)	73 (54%)	138 (38%)	

*AD, Algerian Dinar (1 AD = 0.0070 US$)*.

### History of and Perceptions Toward COVID-19 Infection

The majority of participants (70.0%) declared fearing to contract COVID-19, and 16.0% reported a positive history of COVID-19 infection. On the other hand, only 263 (26.0%) perceived the infection to be severe, while 43.0% believed the disease had no severity. Regarding preventive measures, almost half the participants (48.0%) had a moderate level of adherence, while 43.0% had a high level (Table 1).

### Engagement Toward COVID-19 Vaccine

Overall, we observed low agreement levels regarding vaccine safety (21%), effectiveness (30%), and efficiency to avoid complications (32%). Likewise, a minority declared accepting the COVID-19 vaccine (26%) or willing to take it (21%). Paradoxically, there were lower levels of agreement regarding vaccine safety (14% vs. 25% and 26%), as well as declared acceptance (21% vs 28% and 31%) and willingness (15% vs. 24% and 25%), among healthcare professionals compared with the general population and medical students respectively (*p* < 0.001). Using the engagement score 15 (median) as cutoff, two-thirds of the participants had a low likelihood of engagement (engagement score ≤ 15, 66%) (Table 2).

**Table 2:** Engagement toward COVID-19 vaccine in total population and by comparison between healthcare workers vs. medical students vs. the general population.

**Item/agreement level**	**Total**,	**General population**,	**Healthcare workers**,	**Medical students**,	**P-value**
	***n* (%)**	***n* (%)**	***n* (%)**	***n* (%)**	
I think that SARS-CoV-2 vaccination, whenever available, would be safe					<0.001
Strongly disagree	193 (19%)	113 (22%)	14 (10%)	66 (18%)	
Disagree	136 (13%)	73 (14%)	11 (08%)	52 (14%)	
Neutral	473 (46%)	203 (39%)	75 (55%)	195 (54%)	
Agree	184 (18%)	108 (21%)	29 (21%)	47 (13%)	
Strongly agree	33 (3%)	22 (04%)	7 (05%)	4 (01%)	
I think that SARS-CoV-2 vaccination is effective to prevent infection					0.008
Strongly disagree	150 (15%)	89 (17%)	14 (10%)	47 (13%)	
Disagree	167 (16%)	95 (18%)	19 (14%)	53 (15%)	
Neutral	399 (39%)	179 (34%)	56 (41%)	164 (45%)	
Agree	266 (26%)	131 (25%)	41 (30%)	94 (26%)	
Strongly agree	37 (4%)	25 (5%)	6 (4%)	6 (2%)	
I think that the best way to avoid the complications of COVID-19 is by getting vaccinated					0.005
Strongly disagree	172 (17%)	101 (19%)	12 (09%)	59 (16%)	
Disagree	196 (19%)	103 (20%)	29 (21%)	64 (18%)	
Neutral	319 (31%)	156 (30%)	44 (32%)	119 (33%)	
Agree	268 (26%)	118 (23%)	40 (29%)	110 (30%)	
Strongly agree	64 (6%)	41 (8%)	11 (8%)	12 (3%)	
In principle, I accept to get the SARS-CoV-2 vaccination					<0.001
Strongly disagree	285 (28%)	170 (33%)	21 (15%)	94 (26%)	
Disagree	190 (19%)	78 (15%)	32 (24%)	80 (22%)	
Neutral	279 (27%)	123 (24%)	41 (30%)	115 (32%)	
Agree	201 (20%)	104 (20%)	33 (24%)	64 (18%)	
Strongly agree	64 (6%)	44 (8%)	9 (7%)	11 (3%)	
I will receive the SARS-CoV-2 vaccination as soon as possible whenever it is available					<0.001
Strongly disagree	326 (32%)	181 (35%)	25 (18%)	120 (33%)	
Disagree	195 (19%)	79 (15%)	33 (24%)	83 (23%)	
Neutral	280 (27%)	132 (25%)	43 (32%)	105 (29%)	
Agree	157 (15%)	84 (16%)	29 (21%)	44 (12%)	
Strongly agree	61 (6%)	43 (8%)	6 (4%)	12 (3%)	
Likelihood of engagement					0.145
High (engaged)	342 (34%)	181 (35%)	52 (38%)	109 (30%)	
Low (non-engaged)	677 (66%)	338 (65%)	84 (62%)	255 (70%)	

### Barriers and Enablers of COVID-19 Vaccine Acceptance

**Figure 2:**
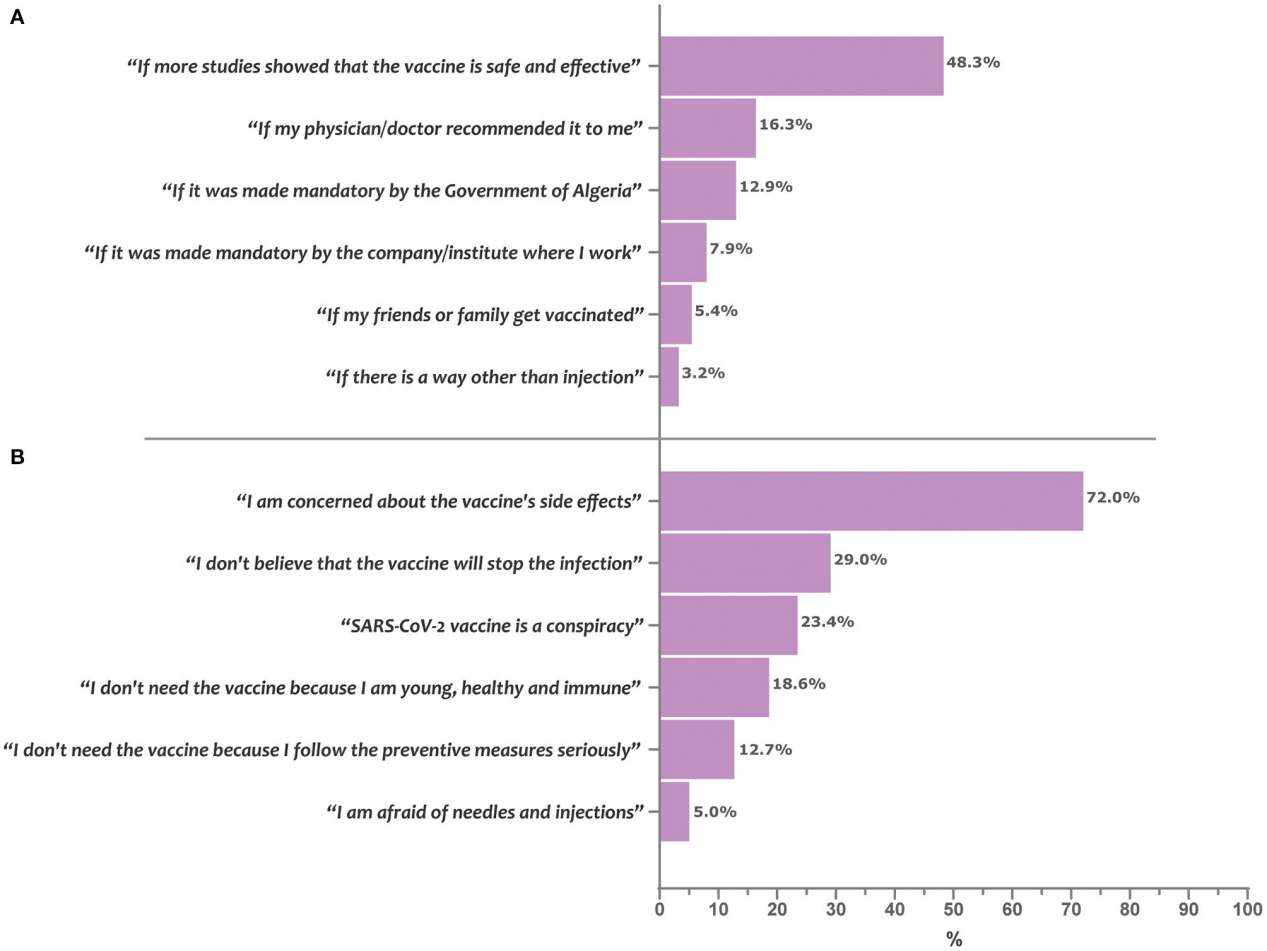
Enablers and barriers of COVID-19 vaccine acceptance in Algeria. Bars represent the percentage of participants who reported the given item as being a determining enabler **(A)** or barrier **(B)** for acceptance of the COVID-19 vaccine uptake.

The barriers and enablers of COVID-19 vaccine acceptance are depicted in Figure 2. Concern about vaccine side effects was the most commonly reported barrier to COVID-19 vaccine acceptance (72.0%), followed by skepticism regarding vaccine efficacy in preventing the infection (29.0%) and beliefs in the conspiracy theory (23.4%). Regarding enablers, exigence for more efficacy and safety studies was the most commonly reported (48.3%), followed by a condition that the vaccine is recommended by the physician (16.3%) or become mandatory (12.9%).

### Factors Associated With COVID-19 Vaccine Non-engagement

In unadjusted models, younger age, female gender, unmarried status, higher income, and higher perceived healthiness; were associated with a higher likelihood for non-engagement to the vaccine, by reference to their respective counterparts. On the other hand, having children, being afflicted with a chronic disease, highly perceived severity of COVID-19, and fear of being infected were associated with a lower likelihood for non-engagement to the vaccine, by reference to their respective counterparts. Further, the level of adherence to preventive measures was inversely associated with non-engagement to the vaccine (Table 3).

**Table 3:** Factors associated with vaccine engagement levels.

**Parameter/category**	**Total (*n* = 1019)**	**Engagement score**	**Non-engagement (Engagement score≤15)**				
	**N (%)**	**Mean ±SD**	**Rate, *N* (%)**	**Unadjusted OR (95%CI)**	**P-value**	Adjusted OR (95%CI)^*^	**P-value**
Age							
More than 60 y	54 (5.3%)	15.91 ± 6.77	23 (42.6%)	Ref		Ref	
40–59 y	107 (10.5%)	13.41 ± 5.96	66 (61.7%)	**2.17 (1.12–4.22)**	**0.023**	1.77 (0.82–3.83)	0.145
30–39 y	208 (20.4%)	13.13 ± 6.08	134 (64.4%)	**2.44 (1.33–4.49)**	**0.004**	1.46 (0.68–3.13)	0.329
18–29 y	650 (63.8%)	13.41 ± 4.57	454 (69.8%)	**3.12 (1.78–5.49)**	**<0.001**	1.39 (0.61–3.17)	0.432
Gender							
Males	474 (46.5%)	13.90 ± 5.74	284 (59.9%)	Ref		Ref	
Female	545 (53.5%)	13.13 ± 4.70	393 (72.1%)	**1.73 (1.33–2.25)**	**<0.001**	**2.31 (1.68–3.18)**	**<** **0.001**
Region							
Center	250 (24.5%)	13.27 ± 5.80	163 (65.2%)	Ref		-	
East	257 (25.2%)	14.14 ± 5.06	158 (61.5%)	0.85 (0.59–1.22)	0.385		
Ouest	252 (24.7%)	13.40 ± 4.99	173 (68.7%)	1.17 (0.81–1.70)	0.411		
South	260 (25.5%)	13.13 ± 4.98	183 (70.4%)	1.27 (0.87–1.84)	0.211		
Area							
Urban	825 (81%)	13.60 ± 5.19	542 (65.7%)	Ref		-	
Rural	194 (19%)	12.98 ± 5.36	135 (69.6%)	1.20 (0.85–1.68)	0.302		
Marital status							
Ever married	307 (30.1%)	13.92 ± 5.92	184 (59.9%)	Ref		Ref	
Never married	712 (69.9%)	13.30 ± 4.88	493 (69.2%)	**1.51 (1.14–1.99)**	**0.004**	1.10 (0.59–2.04)	0.76
Level of education							
Low level	56 (5.5%)	12.20 ± 6.45	37 (66.1%)	Ref		-	
Medium level	110 (10.8%)	13.27 ± 5.88	70 (63.6%)	0.89 (0.46–1.77)	0.757		
High level	853 (83.7%)	13.60 ± 5.03	570 (66.8%)	1.03 (0.58–1.83)	0.908		
House setting							
With family	962 (94.4%)	13.53 ± 5.17	642 (66.7%)	Ref		-	
Alone	57 (5.6%)	12.68 ± 6.04	35 (61.4%)	0.79 (0.46–1.37)	0.408		
Living with someone who has a chronic disease							
No	488 (47.9%)	13.26 ± 5.17	334 (68.4%)	Ref		-	
Yes	531 (52.1%)	13.70 ± 5.26	343 (64.6%)	0.84 (0.65–1.09)	0.194		
Having chronic disease							
No	883 (86.7%)	13.34 ± 5.15	598 (67.7%)	Ref		Ref	
Yes	136 (13.3%)	14.45 ± 5.57	79 (58.1%)	**0.66 (0.46–0.96)**	**0.027**	0.88 (0.56–1.38)	0.579
Job							
Unemployed	144 (14.1%)	12.76 ± 5.86	100 (69.4%)	Ref		Ref	
Healthcare sector	136 (13.3%)	14.66 ± 4.61	84 (61.8%)	0.71 (0.43–1.17)	0.177	0.60 (0.32–1.02)	0.057
Public sector	165 (16.2%)	13.60 ± 5.57	106 (64.2%)	0.79 (0.49–1.27)	0.334	0.80 (0.46–1.40)	0.438
Privat sector	122 (12%)	13.84 ± 6.24	70 (57.4%)	**0.59 (0.36–0.98)**	**0.042**	**0.53 (0.29–0.97)**	**0.038**
Student	364 (35.7%)	13.20 ± 4.61	255 (70.1%)	1.03 (0.67–1.57)	0.892	0.67 (0.39–1.15)	0.147
Others	88 (8.6%)	13.34 ± 4.99	62 (70.5%)	1.05 (0.59–1.87)	0.871	1.07 (0.55–2.06)	0.851
Income							
>100K AD	199 (19.5%)	14.69 ± 5.22	117 (58.8%)	Ref		Ref	
50K−100K AD	347 (34.1%)	13.48 ± 5.02	235 (67.7%)	**1.47 (1.03–2.11)**	**0.036**	1.47 (0.99–2.17)	0.051
<50K AD	473 (46.4%)	12.98 ± 5.29	325 (68.7%)	**1.54 (1.09–2.17)**	**0.014**	1.34 (0.92–1.95)	0.132
Children							
No	763 (74.9%)	13.30 ± 4.96	527 (69.1%)	Ref		Ref	
Yes	256 (25.1%)	14.04 ± 5.92	150 (58.6%)	**0.63 (0.47–0.85)**	**0.002**	0.73 (0.40–1.35)	0.315
Fear of getting the disease							
No	144 (14.1%)	11.12 ± 5.37	116 (80.6%)	Ref		Ref	
Got the disease	164 (16.1%)	13.70 ± 4.58	114 (69.5%)	**0.55 (0.32–0.94)**	**0.027**	0.68 (0.38–1.21)	0.19
Yes	711 (69.8%)	13.92 ± 5.21	447 (62.9%)	**0.41 (0.26–0.63)**	**<** **0.001**	**0.56 (0.35–0.91)**	**0.018**
Perception of COVID-19 severity							
Null	439 (43.1%)	12.36 ± 5.50	318 (72.4%)	Ref		Ref	
Medium	317 (31.1%)	14.38 ± 4.79	194 (61.2%)	**0.60 (0.44–0.82)**	**0.001**	0.76 (0.52–1.09)	0.134
High	263 (25.8%)	14.30 ± 4.90	165 (62.7%)	**0.64 (0.46–0.89)**	**0.007**	**0.66 (0.47–0.92)**	**0.014**
Health perception							
Below average	131 (12.9%)	14.17 ± 5.67	76 (58.0%)	Ref		Ref	
Good/excellent	888 (87.1%)	13.39 ± 5.15	601 (67.7%)	**1.52 (1.04–2.20)**	**0.03**	1.45 (0.94–2.24)	0.097
Level of Adherence to preventive measures							
High level	435 (42.7%)	15.06 ± 5.24	243 (55.9%)	Ref		Ref	
Medium level	491 (48.2%)	12.78 ± 4.79	352 (71.7%)	**2.00 (1.52–2.63)**	**<0.001**	**2.07 (1.54–2.78)**	**<0.001**
Low adherence	93 (9.1%)	9.86 ± 4.74	82 (88.2%)	**5.89 (3.05–11.36)**	**<** **0.001**	**6.93 (3.46–13.87)**	**<0.001**

*95% CI, 95% confidence interval; AD, Algerian dinar; N, number; OR, odds ratio; SD, standard deviation; y, years;*

* *adjusted for age gender marital status having chronic disease job income having children fear from getting the disease perception of severity of the disease health perception and level of adherence to preventive measures. Bold value indicates statistical significance*.

**Figure 3:**
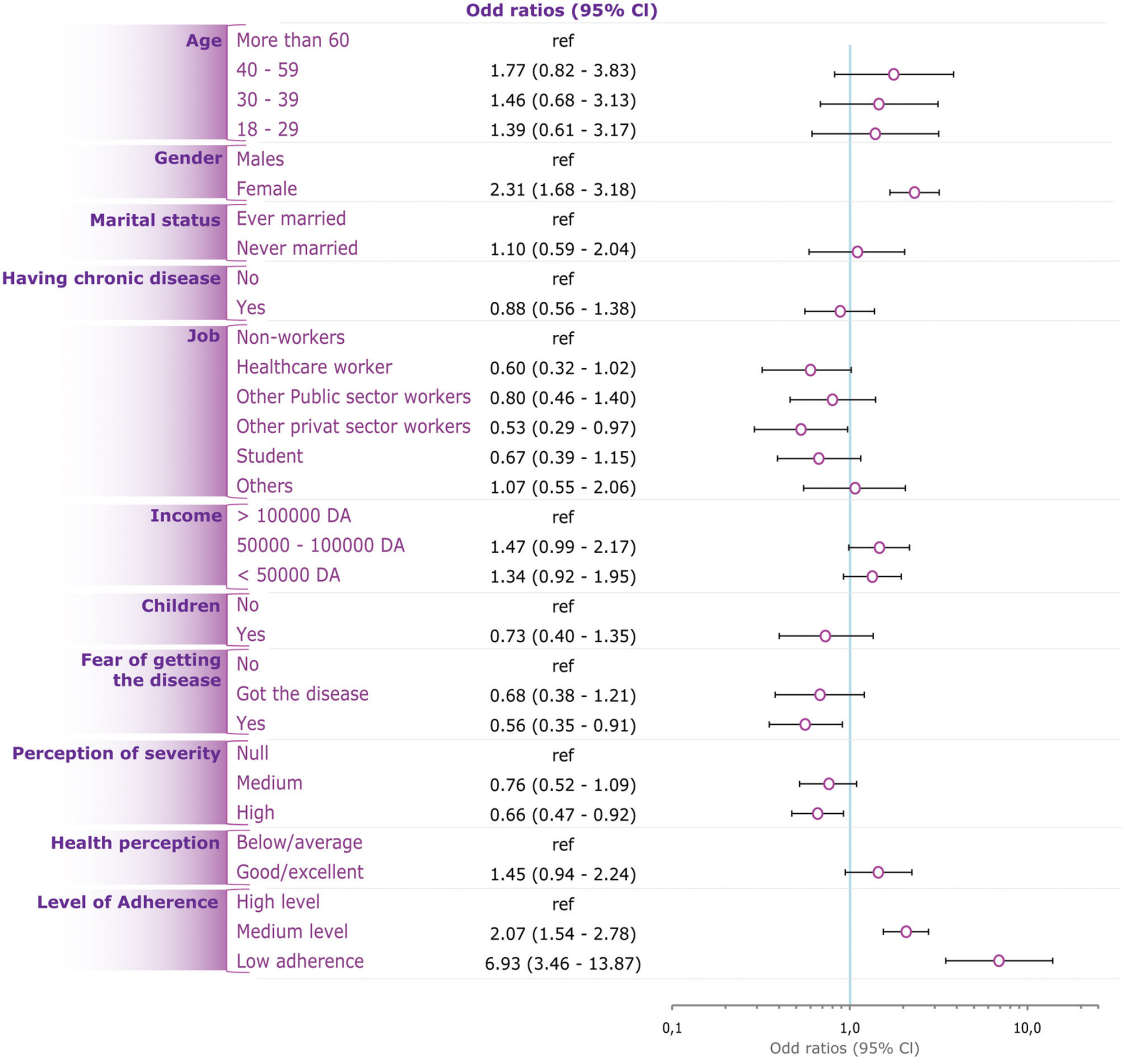
Predictors of nonengagement to COVID-19 vaccine in Algeria.

The Adjusted model showed that the likelihood for non-engagement was independently associated with female gender (OR = 2.31; 95%CI: 1.68–3.18, *p* <0.001), medium (OR = 2.07, 95%CI: 1.54–2.78, *p* <0.001) and low adherence level to preventive measures (OR = 6.93; 95%CI: 3.46–13.87, *p* <0.001), work in private sector (OR = 0.53; 95%CI: 0.29–0.97, *p* = 0.038), high perceived COVID-19 severity (OR = 0.66; 95%CI: 0.47–0.92, *p* = 0.014), and fear from contracting the disease (OR = 0.56; 95%CI: 0.35–0.91, *p* = 0.018) (Table 3, Figure 3).

### COVID-19 Acceptance in Arab Countries From MENA Region—Results of the Systematic Review

A total of six studies were included in this systematic review, with sample sizes ranging from 1,019 to15,087 participants. Eleven studies were excluded, out of which six were not conducted among the general population, and five studies from the same countries comprised a smaller sample size as shown in Figure 1. The included studies were conducted in the United Arab Emirates (UAE), Kuwait, Qatar, Libya, Kingdom of Saudi Arabia (KSA), and Jordan (Table 4). All studies were internet-based, nationwide surveys; three studies (32–34) were conducted only amongst the general population, while the remaining comprised the general population and healthcare workers (31, 32, 36). The quality ranking of the included cross-sectional studies across different criteria is reported in the (Supplementary Table 3) a green color for “yes,” red for “no,” grey for not applicable and yellow for “cannot determine” respectively. The overall quality was considered as fair for all the studies. The highest COVID-19 vaccine acceptance rate (75%) was reported in UAE (32), followed by Kuwait (65%) (33), Qatar (61%) (35), and Libya (61%) (36). Predictors of vaccine acceptance varied between the studies, and included adherence to government recommendations, married status, positive COVID-19 status, having friends died or infected with COVID-19, high income, fear of contracting COVID-19, perception of high severity, and private-sector workers. History of flu vaccination was a positive predictor of COVID-19 vaccination in three studies by Alabdulla et al. (35), Alfageeh et al. (34), and El-Elimat et al. (39). Female gender was a significant predictor for vaccine avoidance in the study by Alfageeh et al. (34). Other vaccine avoidance predictors that were reported comprised younger age, self-employment, safety concerns, conspiracy theory, and low and medium adherence to COVID-19 preventive measures.

**Table 4:** Characteristics of studies included from the MENA regions.

**References**	**Country**	**Study** **Period**	**Setting/** **population**	**Sampling & recruitment**	**Sample size**	**Males, *n* (%)**	**Older age category, *n* (%)**	**Acceptance rate**	**Predictors for acceptance**	**Predictors for avoidance**
Muqattash et al. (32)	United Arab Emirates	04/07/2020 04/08/2020	National, population-based	Snowball sampling, Web-based	1,109	309 (28%)	>45 y, 219 (20%)	75%	NA	NA
AlAwadhi et al. (33)	Kuwait	16/05/2020 31/08/2020	National, population-based	Convenient sampling, Web-based	5,651	1,321 (23%)	>60 y, 382 (7%)	65%	High adherence to recommendations by the government.	Female gender, Younger age, Ever married.
Alabdulla et al. (35)	Qatar	15/10/2020 15/11/2020	National, population-based including HCWs	Convenient sampling, Web-based	7,821	4,648 (59%)	>65 y, 325 (4%)	61%	Ever married, Flu vaccination.	Female gender, Younger age, Self-employment, Safety concerns.
Elhadi et al. (36)	Libya	01/12/2020 18/12/2020	National, population-based including HCWs	Snowball sampling, Web-based	15,087	6,227 (41%)	>50 y, 675 (5%)	61%	Currently infected with COVID-19, Having a friend infected/died from COVID-19.	Younger age, Never married.
Alfageeh et al. (34)	Saudi Arabia	08/12/2020 14/12/2020	National, population-based	Snowball sampling, Web-based	2,137	1,227 (57%)	>60 y, 212 (10%)	48%	Fear from being infected, High income, Flu vaccination.	Female gender.
El-Elimat et al. (39)	Jordan	01/11/2020 01/12/2020	National, population-based including HCWs	Convenient sampling, Web-based	3,100	1,012 (33%)	>35 y, 1,060 (34%)	37%	Flu vaccination.	Female gender, Younger age, Employment, Conspiracy theory, Safety concerns.

## Discussion

This is the first nationwide study addressing the Algerian population's attitude toward the COVID-19 vaccine. Using a multidimensional model to measure the likelihood of engagement to vaccination, our study revealed that only 34% of the participants would be engaged to receive the COVID-19 vaccines. The Adjusted regression analysis demonstrated multiple predictors for non-engagement, including female gender, and low/intermediate levels of adherence to preventive measures, whereas a high perception of the disease severity and fear of being infected predicted vaccine acceptance. Additionally, the systematic review findings suggested that Algeria had the lowest vaccine acceptance rate in comparison with other MENA countries, where acceptance rates ranged from 37.4% in Jordan (39) and 75% in the UAE (32). More recent data showed greater disparity in vaccine acceptance rates in the MENA region (23). In comparison with Europe, the lowest acceptance rate of 53.7%, reported in Italy (40), was relatively higher than the acceptance rate observed in our study.

The high perceived severity of COVID-19 was among the independent risk factors for engagement; however, only 25.8% of participants perceived the disease to be severe. Regardless of the acceptability of the vaccine, the severity of the disease will affect the vaccination intention. Perception about the disease severity may be assimilated to a personal opinion or belief regarding the level of hazard or exposure to the crisis and the extent of its adverse impact on the individual (41). In the case of COVID-19, but not specifically, the risk perception may change over time and is further determined by the individual's awareness about and interpretation of the relationship between the virus/pandemic and the observed undesirable effects—and such interpretation may be biased or distorted by other opinions, (mis)beliefs and (mis)conceptions. A theoretical approach by Cori et al. (42), suggested that both risk perception and fear of COVID-19 are determined by cognitive factors, and the author mentioned four key factors including knowledge about the disease/virus, visibility of the risk, trust in the authorities, and healthcare institutions, and voluntary exposure to the virus/infection. The aforementioned factors may be modified by means of awareness-raising campaigns and authoritarian corrective or restrictive measures, aiming at enhancing the risk perception and ultimately increasing the vaccination rates. Evidence from previous data suggests that risk perception about COVID-19 increased in the lockdown phase and decreased in the re-opening phases (43), which was positively associated with the change in vaccine acceptance rate. At the time when the present study was conducted, the country was in a post-re-opening phase, which may explain the low engagement rates observed. Another longitudinal study from the US assessed the trend of people's attitude toward the vaccine, between March and August 2020, and showed heterogeneous results with perceived severity of the disease being one of the determinants of the vaccine acceptance. Furthermore, the authors demonstrated that the trends in both risk perception and vaccine acceptance were likely to be determined by the individual's specific political positions and exposure to media (44). Such observation supports the importance of correcting the cognitive and behavioral factors at the population's level to enhance vaccine uptake.

Similar to other reports from the MENA region, including Kuwait (33), Qatar (35), KSA (34), and Jordan (39), men were more likely to accept the Covid-19 vaccine in Algeria. This can be explained by the increased severity of the disease among men and the higher mortality reported in the majority of countries (45, 46). This statement was extensively mediatized and may have played a role in men's motive to vaccination, developing a relatively more positive attitude toward the vaccine. While such an explanation requires further evidence, notably the associated levels of awareness about the specific health risks on males, other factors may explain the less negative attitude among males that was found in the present study. Among these factors, the impact of the pandemic and restrictive measures on incomes and businesses, which may be more perceived by males in some societies. This explanation may be in line with the significant association of vaccine engagement with being married and having children that were found in the unadjusted analysis in the present study. Another potential factor explaining this gender disparity is the belief that COVID-19 is part of a global conspiracy, which was reportedly more common in women, thus explaining the higher vaccine hesitancy of females in some populations (21, 47).

However, past research data showed conflicting results about gender. A global survey including 13,426 individuals in 19 countries with a high COVID-19 burden showed that men were relatively less likely to have a positive attitude toward vaccination than women (48). Another study showed that women in Russia and Germany had higher acceptance rates of the COVID-19 vaccines than men (49). This phenomenon has been named “*the* Covid-19 *gender paradox*” (50). This gender difference can be explained by multidimensional psychological, social, cultural, and environmental influences. Further research may be required to determine the gender-specific factors associated with acceptance or refusal of the vaccine, which would enable designing targeted awareness campaigns with gender-specific messages to enhance the vaccine acceptance rates in both genders.

There is a remarkable similarity between the engagement rates of the general population (35%) and healthcare workers (38%) in the present study, which is an issue of big concern as it may constitute a significant barrier to the national vaccine campaign. Indeed, the practitioner's vaccine hesitancy influences the vaccination attitudes of the patients (51). When providers are unsure of the safety of the vaccine, they are unable to recommend it to the general population. Such an issue should be considered at the critical level by the health authorities, and corrective measures are warranted urgently to increase awareness among health providers. Furthermore, this study showed comparable patterns of safety concerns about the vaccine in the two subgroups, i.e., health workers vs. the general population (75% and 73%, respectively). This indicates the consistency of the popular misconceptions about the COVID-19 vaccine across all categories of the studied population and highlights the need for a comprehensive awareness-raising campaign at the national scale.

Other notable factors of vaccine refusal include fear of the side effects and concern about the efficiency of the vaccines. Similar concerns have been reported in other countries such as Jordan (39, 52) and the USA (52). Arguably, these concerns may be comprehensible, considering the rapid vaccine development process, the novelty of the mRNA technology used in some vaccines, and the public mediatization of the vaccine side effects; all exposing the population to massive misinformation notably in the social media (53, 54). This could be related to the decreasing acceptance rate over time in the MENA region as shown in the systematic review part of the study. Hence there is a crucial need to implement effective strategies to correct the popular misconceptions regarding the vaccine's safety.

This study also highlighted the positive association between the level of adherence to preventive measures and vaccine acceptability. This observation is in accordance with another MENA region study in Kuwait (33), reporting that high adherence to the governmental recommendations was an important predictor for vaccine uptake. Both low adherence to preventive measures and adverse attitudes toward vaccines could reflect adherence to the conspiracy theory, and this was observed among 23.4% of the avoidant group. Conspiracy theories have been associated with vaccine hesitancy as a result of mistrust between the public and the government policymakers (50).

## Strengths and Limitations

One of the strengths of the present study is the use of a multidimensional model to define vaccine engagement based on a conceptual framework combining perceived vaccine effectiveness and safety with self-declared acceptance and willingness. This combination is assumed to be more reliable than using self-declared acceptance and willingness, as perceived safety and efficacy are less subjected to social desirability bias. Yet, the scale requires further validation to support this assumption. On the other hand, there are no validated instruments to assess attitudes toward the COVID-19 vaccines, and the relevant studies principally used various formulations of self-declared willingness or preparedness, which is limited by the high risk of negative or positive social desirability bias. Future research is recommended in this regard to design a validated scale to measure vaccine acceptance based on a strong model, which will enhance the quality and comparability of the findings. Another strong point of this study is that participants were equally distributed from the 4 regions of the country, which supports the generalizability of the findings. Further, determinants of vaccine acceptance and avoidance were highlighted for the first time nationwide. Therefore, the findings of this study can have a high impact on health authorities' decisions for the management of vaccination campaigns.

The major limitation of this study is the recruitment method of the participants, which was restricted to those who have access to the internet and an electronic device since the questionnaire was shared online. This probably led to a selection bias, occulting a non-negligible section of the population that may have distinct characteristics. One of these characteristics is the source of information regarding COVID-19 disease and vaccine, which may be radically different in the subpopulation of internet non-users by reference to internet users. This may result in discrepant opinions and attitudes toward the vaccine by reference to the study population. Unfortunately, no data was collected about sources of information about the vaccines, which would provide an indication about the aforementioned issue. Nevertheless, a study showed that individuals who get information from the internet are less inclined to accept the COVID-19 vaccine than those who get information from healthcare workers (55). Another aspect of the selection bias is the overrepresentation of medical students and healthcare providers, which was probably due to the snowball sampling method and which limits the generalizability of the findings.

## Conclusion

Two-third of Algerians are likely to be non-engaged for COVID-19 vaccine uptake, making them one of the least accepting public for the voluntary vaccination in the MENA region. This probably provides an explanation for the slow ascension of the vaccination curve, which constitutes a great public health concern. These findings and their interpretation should be taken into consideration by the policymakers to acknowledge and address the adverse attitude about the vaccine, notably among healthcare providers who are the vectors and major contributors of a successful vaccine policy. Vaccine awareness campaigns should be implemented to address the multiple misconceptions and enhance the levels of knowledge and perception both about the disease and the vaccine, by prioritizing target populations and engaging both healthcare workers and the general population.

## Data Availability Statement

The original contributions presented in the study are included in the article/Supplementary Material, further inquiries can be directed to the corresponding author/s.

## Author Contributions

SK, SK-D, FY, ME, and MH were responsible for the idea and study design. SK analyzed the data under the supervision of MH. SK, SK-D, FY, and MH wrote the first draft of the manuscript. SG, JS, and MH critically revised the original draft. All authors collected the data, performed systematic review screening and extraction, interpreted the data, shared in the writing, formatting, and approval of the final version.

## Conflict of Interest

The authors declare that the research was conducted in the absence of any commercial or financial relationships that could be construed as a potential conflict of interest.

## Publisher's Note

All claims expressed in this article are solely those of the authors and do not necessarily represent those of their affiliated organizations, or those of the publisher, the editors and the reviewers. Any product that may be evaluated in this article, or claim that may be made by its manufacturer, is not guaranteed or endorsed by the publisher.

## Abbreviations

AD: Algerian Dinars
KSA: Kingdom of Saudi Arabia
MENA: Middle-East and North African
OR: Odds ratio
PRISMA: Preferred Reporting Items for Systematic Reviews and Meta-Analyses
SD: standard deviation
STROBE: Strengthening the Reporting of Observational Studies in Epidemiology
UAE: United Arab Emirates.
