# Supplementary material for: Determinants of COVID-19 Vaccine Engagement in Algeria: A Population-Based Study With Systematic Review of Studies From Arab Countries of the MENA Region
Source: Front Public Health. 2022 May 30;10:843449. doi: 10.3389/fpubh.2022.843449 (PMC9196869; doi:10.3389/fpubh.2022.843449)
Supplement: Supplementary file 1 [file Data_Sheet_1.pdf]

Exclusion criteria & Consent

1) Have you been vaccinated against the COVID-19?
   - Yes
   - No
2) Do you agree to participate in this study?
   - Yes
   - No

General information

1) Your age category?
   - 18 - 29
   - 30 - 39
   - 40 - 59
   - > 60
2) Are you?
   - Male
   - Female
3) Your job?
   - Unemployed
   - I work in the healthcare sector
   - I work in the public sector
   - I work in the privat sector
   - I am a student
   - Others
4) Level of education?
   - No education
   - Primary school
   - Middle school
   - High school
   - Higher education without diploma
   - Higher education with diploma
5) Family income?
   - > 100K AD
   - 50K – 100K AD
   - < 50K AD
6) Region of residency?
   - North
   - South
   - East
   - West
7) Do you live in?
   - Rural area
   - Urban area
8) Do you live?
   - Alone
   - with family
9) Marital status?
   - Ever married
   - Never married
10) Do you have children?
   - Yes
   - No
11) Do you have a chronic disease?
   - Yes
   - No
12) Do you live with someone with chronic diseases?
   - Yes
   - No
13) What is your assessment of your physical wellbeing/health?
   - Below average

- Average
- Good
- Excellent

14) What is your assessment of the possibility of infection with the COVID-19 during the next six months?
- I got the disease
- Null probability
- Weak probability
- High probability

15) To what extent do you worry about catching a severe form of COVID-19 in the next six months?
- I am not worried about that at all
- That worries me a bit
- That worries me somewhat
- It worries me a lot

Adherence to preventive measurement

How committed were you to the following preventive measures during the last three months?

1) Face mask when going out
- Never
- Rarely
- Sometimes
- Often
- Always

2) Not touching the face and the mask
- Never
- Rarely
- Sometimes
- Often
- Always

3) Clean hands when entering the house
- Never
- Rarely
- Sometimes
- Often
- Always

4) Covering the face when coughing or sneezing
- Never
- Rarely
- Sometimes
- Often
- Always

5) Not shaking hands/kissing friends
- Never
- Rarely
- Sometimes
- Often
- Always

6) Maintaining a safe distance
- Never
- Rarely
- Sometimes
- Often
- Always

7) Staying at home in case of illness
- Never
- Rarely
- Sometimes
- Often

- Always

8) If I have a fever, cough and difficulty breathing, seek medical attention.
    - Never
    - Rarely
    - Sometimes
    - Often
    - Always

About COVID-19 vaccine

Based on your personal view, to what extent do you agree with the following points?

1) I think that COVID-19 vaccination, whenever available, would be safe
    - Strongly disagree
    - Disagree
    - Neutral
    - Agree
    - Strongly agree
2) I think that COVID-19 vaccination is effective to prevent infection.
    - Strongly disagree
    - Disagree
    - Neutral
    - Agree
    - Strongly agree
3) I think that the best way to avoid the complications of COVID-19 is by getting vaccinated.
    - Strongly disagree
    - Disagree
    - Neutral
    - Agree
    - Strongly agree
4) In principle, I accept to get the COVID-19 vaccination.
    - Strongly disagree
    - Disagree
    - Neutral
    - Agree
    - Strongly agree
5) I will receive the COVID-19 vaccination as soon as possible whenever it is available
    - Strongly disagree
    - Disagree
    - Neutral
    - Agree
    - Strongly agree

Enablers and Barriers

Mark all of the following factors that prevent you from getting the COVID-19 vaccination?

- I am concerned about the vaccine's side effects
- I don't believe that the vaccine will stop the infection
- COVID-19 vaccine is a conspiracy
- I don't need the vaccine because I am young, healthy and immune
- I don't need the vaccine because I follow the preventive measures seriously
- I am afraid of needles and injections

Among the following factors, what would encourage you to get vaccinated against COVID-19?

- If more studies showed that the vaccine is safe and effective
- If my physician recommended it to me
- If it was made mandatory by the government of Algeria
- If it was mandatory by the company/institute where I work
- If my friends or family get vaccinated
- If there is a way other than injection
