# Supplementary material for: Determinants of COVID-19 Vaccine Engagement in Algeria: A Population-Based Study With Systematic Review of Studies From Arab Countries of the MENA Region
Source: Front Public Health. 2022 May 30;10:843449. doi: 10.3389/fpubh.2022.843449 (PMC9196869; doi:10.3389/fpubh.2022.843449)
Supplement: Supplementary file 2 [file Table_1.pdf]

Supplemental Table 1: Inter-correlation matrix of the variables used in the calculation of engagement score

	Item 1	Item 2	Item 3	Item 4	Item 5
Item 1	1.00	0.70	0.68	0.79	0.80
Item 2		1.00	0.89	0.75	0.77
Item 3			1.00	0.72	0.74
Item 4				1.00	0.78
Item 5					1.00

Item 1: I think that SARS-CoV-2 vaccination, whenever available, would be effective.
Item 2: In principle, I accept to get the SARS-CoV-2 vaccination.
Item 3: I will receive the SARS-CoV-2 vaccination as soon as possible whenever it is available.
Item 4: I think that the best way to avoid the complications of COVID-19 is by getting vaccinated.
Item 5: I think that SARS-CoV-2 vaccination, whenever available, would be safe.
Cronbach Alpha = 0.94.
