# Supplementary material for: Determinants of COVID-19 Vaccine Engagement in Algeria: A Population-Based Study With Systematic Review of Studies From Arab Countries of the MENA Region
Source: Front Public Health. 2022 May 30;10:843449. doi: 10.3389/fpubh.2022.843449 (PMC9196869; doi:10.3389/fpubh.2022.843449)
Supplement: Supplementary file 3 [file Table_2.pdf]

Supplemental Table 2: Inter-correlation matrix of the variables used in the calculation of adherence score

	Item 1	Item 2	Item 3	Item 4	Item 5	Item 6	Item 7
Item 1	1.00	0.43	0.55	0.34	0.51	0.47	0.38
Item 2		1.00	0.37	0.58	0.32	0.39	0.33
Item 3			1.00	0.32	0.59	0.46	0.37
Item 4				1.00	0.35	0.43	0.42
Item 5					1.00	0.55	0.40
Item 6						1.00	0.54
Item 7							1.00

Item 1: I don’t touch my nose, ears or mouth.
Item 2: I clean my hands using soap and water, or alcohol-based hand rub.
Item 3: I avoid shaking hands when I am outside.
Item 4: I cover my nose and mouth with my bent elbow or a tissue when I cough or sneeze.
Item 5: I maintain safe distance from anyone who is coughing or sneezing.
Item 6: I stay at home when I feel unwell.
Item 7: I seek medical attention when I have fever, cough and difficulty breathing.
Cronbach Alpha = 0.84.
